# Effect of faba bean-based diets on the meat quality and fatty acids composition in breast muscles of broiler chickens

**DOI:** 10.1038/s41598-020-62282-7

**Published:** 2020-03-24

**Authors:** Joanna Kuźniacka, Mirosław Banaszak, Jakub Biesek, Giuseppe Maiorano, Marek Adamski

**Affiliations:** 1 Department of Animal Breeding, Faculty of Animal Breeding and Biology, UTP - University of Science and Technology in Bydgoszcz, Mazowiecka 28, 85-084 Bydgoszcz, Poland; 2 Department of Agricultural, Environmental and Food Sciences, University of Molise, Via De Sanctis snc, 86100 Campobasso, Italy

**Keywords:** Biophysical chemistry, Zoology

## Abstract

The aim of the study was to evaluate the effect of feed containing faba bean on the physicochemical properties of breast and leg muscles. The study was conducted on 340 Ross 308 broiler chickens reared for 6 weeks. The control group received feed based on soybean meal. The treatment group received a feed mixture with faba bean as the source of protein. Different sources of protein in the diet were also associated with changes in the content of n-6 fatty acids (C16:0, C22:4) and the n-6/n-3 ratio in breast muscles, which was higher (P < 0.05) in treatment group. The collagen content was higher (P < 0.05) in breast muscles from control group. The study revealed that the use of faba bean as a substitute for soybean meal had no significant effect (P > 0.05) on water holding capacity, drip loss, or major chemical components of breast and leg muscles. The P/S ratio, AI and TI, and the content of cholesterol in breast muscles were comparable (P > 0.05) in both groups. The values of lightness (L*) for leg muscles were lower (P < 0.05) in treatment group. The use of faba bean instead to soybean meal in diets for broiler chickens had positive effects on meat quality traits.

## Introduction

Consumers consider the safety and high quality of products to be essential aspects of poultry production. Diet, in addition to genotype, age and sex of birds and their management system, is one of the most important factors determining the quality of poultry meat^1,2^. The use of an appropriate compound feed allows for the proper growth of birds, which is also associated with more and more frequently assessed welfare. Not only the genotype used for intensive broiler production plays a role. An important element is the adaptation of production technology, which includes the environment, maintenance time, nutrition. It all consists of welfare^3,4^. In most countries imported soybean meal, usually produced from genetically modified plants, is the main source of protein in feeds for broiler chickens. This considerably increases the cost of broiler chicken production, where expenditure on feed may comprise as much as 70% of the total costs. Moreover, consumers are often concerned about GMO products^5–7^. Chicken meat has excellent nutritional value since it is rich in highly digestible protein, which includes all exogenous amino acids. It is low in fat and has excellent taste properties. Chicken meat is cheap compared to other meat types, it is easily portioned, and is quick and easy to cook. Other aspects of meat quality include its safety for human health, nutritional value, functional properties and sensory properties^8–15^.

The improvement of feed mixtures is in mainstream research into the cost-effectiveness of broiler chicken production. The profitability of production of all poultry species largely depends on soybean meal. The use of genetically modified soybean in Poland is allowed under the Animal Feed Act, which in the near future is expected to ban the import and distribution of feed from genetically modified plants. Soybean is a plant with specific climatic requirements, and its cultivation is possible and profitable only in certain regions, which is why countries with an unsuitable climate are dependent on imported SBM. Because of this, legume plants, and their nutritional value and potential use for the formulation of poultry feed have attracted growing interest in recent years^1,7,16–18^. Faba bean is high-protein plant. New cultivars characterized by low content of tannins^19^. The authors concluded that faba bean seeds are good source of protein. No negative effect on carcass traits of turkeys were found by Przywitowski *et al*.^20^. Even 31% of faba beans in diet had no negative effect on broiler growth performance and meat quality^21^.

The testes hypothesis is: The use of faba bean seeds in diets for broiler chicken has an effect on the quality of chicken meat and fatty acids profile in breast muscles.

The aim of the study was to analyze the physicochemical properties, major chemical components, and fatty acids composition of meat from broiler chickens fed faba bean as a source of protein alternative to soybean meal.

## Results

There were no significant differences (P > 0.05) in the values of pH_24_ for breast muscles between chickens fed soybean meal and faba bean (Table 1). However, pH_15_ measured for breast muscles from group 2 (diet based on faba bean) was significantly higher compared to group 1 (soybean meal) (5.96 vs 5.76). Values of colour parameters for breast muscles (L*, a*, b*), water holding capacity and drip loss were comparable in both groups. Regardless of the source of protein in chicken diet, breast muscles did not differ significantly for the content of protein (24.23–24.26%), fat (1.39–1.86%) or water (73.74–73.89%) (Table 2). The analysis of the chemical composition of leg muscles also showed no significant differences in the content of protein, fat or water (Table 3). However, the lightness of leg muscles (L*) differed depending on the source of protein in feed mixtures. The value of lightness (L*) of leg muscles from birds fed faba bean was significantly higher compared to birds fed soybean meal (50.22 vs 48.38).

**Table 1:** Physicochemical parameters (means, SEM) of breast muscles from 6-week-old chickens.

Group^1^ n = 10	pH_15_	pH_24_	Colour			Water holding capacity (%)	Drip loss (%)	Protein (%)	Fat (%)	Water (%)
			L*	a*	b*					
1	5.76^b^	5.88	50.06	2.10	7.70	35.91	0.85	24.26	1.86	73.89
2	5.96^a^	5.93	49.71	2.90	7.37	34.03	0.82	24.23	1.39	73.74
SEM	0.04	0.02	0.65	0.25	0.28	1.01	0.14	0.00	0.06	0.02
*P*-value	0.005	0.286	0.792	0.109	0.677	0.370	0.908	NS	NS	NS

^1^ – 1 = feed based on soybean meal, 2 = feed based on faba bean (*Vicia faba* var. minor), n – number of birds taken to analysis.

a, b… – means in columns marked with different letters differ significantly between groups, p-value < 0.05, NS – no significant.

**Table 2:** Fatty acids composition (% of total fatty acids) in breast muscles from 6-week-old chickens.

Fatty acid^2^	Group^1^ n = 10		SEM	*P*-value
	1	2		
C14:0	0.35	0.28	0.02	0.032
C16:0	20.97^B^	22.46^A^	0.25	0.001
C16:1n-7	1.91	1.48	0.14	0.129
C18:0	10.03	10.66	0.51	0.549
C18:1n-9	27.12	28.94	0.88	0.317
C18:2 n-6	28.23	23.84	0.77	0.002
C18:3 n-3	1.97	1.52	0.14	0.118
C20:1n-9	0.83	0.36	0.06	0.000
C20:4 n-6	5.56	8.50	0.60	0.010
C20:5 n-3	0.22	0.24	0.02	0.733
C22:4 n-6	0.34^B^	0.69^A^	0.06	0.001
C22:5 n-3	1.18	0.69	0.09	0.003
C22:6 n-3	0.56	0.35	0.05	0.037
ΣSFA	31.35	33.40	0.62	0.098
ΣMUFA	29.86	30.78	0.97	0.648
ΣPUFA	38.05	35.82	0.68	0.102
Total n-6	34.13	33.03	0.58	0.358
Total n-3	3.93	2.79	0.17	0.000
n-6/n-3	8.74^b^	12.43^a^	0.70	0.005
P/S	1.21	1.08	0.03	0.008
AI	0.33	0.35	0.01	0.041
TI	0.72	0.83	0.02	0.012

^1^ – 1 = feed based on soybean meal, 2 = feed based on faba bean (*Vicia faba* var. minor), n – number of samples taken to analysis.

^2^ – SFA = saturated fatty acids, MUFA = monounsaturated fatty acids, PUFA = polyunsaturated fatty acids, P/S = PUFA/SFA ratio, AI = atherogenic index; TI = thrombogenic index.

a, b… – mean values marked in columns with different letters differ significantly between groups, *P*-value <0.05; A, B… – mean values marked in columns with different letters differ significantly between groups, *P*-value <0.01.

**Table 3:** Physicochemical parameters (means, SEM) of leg muscles from 6-week-old chickens.

Group^1^ n = 10	Colour			Water holding capacity (%)	Protein (%)	Fat (%)	Water (%)
	L*	a*	b*				
1	48.38^b^	3.81	7.88	33.33	20.82	5.72	72.28
2	50.22^a^	5.64	6.18	37.71	19.92	6.17	72.29
SEM	0.48	0.78	0.81	1.28	0.12	0.06	0.00
*P*-value	0.050	0.253	0.311	0.086	NS	NS	NS

^1^ – 1 = feed based on soybean meal, 2 = feed based on faba bean (*Vicia faba* var. minor), n – number of birds taken to analysis.

a, b… – means in columns marked with different letters differ significantly between groups, p-value < 0.05, NS – no significant.

Data in Table 2 indicate that the use of different sources of protein in the diet of broiler chickens influenced the content of C16:0 fatty acid in breast muscles, which was significantly higher (P < 0.01) in group 2 fed faba bean than in group 1 fed SBM (22.46% vs 20.97% of total fatty acids). Muscles from group 2 were characterised by significantly higher (P < 0.01) content of C22:4 n-6 fatty acid (0.69%) and higher (P < 0.05) n-6/n-3 ratio (12.43) compared to group 1, where these values were 0.34% and 8.74, respectively. The ratio of polyunsaturated fatty acids to saturated fatty acids (P/S) was comparable in both groups (1.08 in group 1 vs 1.21 in group 2). The values of atherogenic index (AI) were also similar (0.33 in group 1 vs 0.35 in group 2). The thrombogenic index (TI) was slightly higher in chickens fed faba bean (0.83) than in chickens fed soybean meal (0.72), but the difference was not significant.

There was no significant difference in the content of cholesterol in breast muscles from both groups (Table 4), but a significant difference was found in the content of collagen in breast muscles (P < 0.01). The content of collagen was significantly higher in breast muscles from group 1 (28.95%) compared to group 2 (20.27%).

**Table 4:** Content of cholesterol (mg/100 g) and collagen (µg/mg of lyophilized muscle tissue) in breast muscles from 6-week-old chickens.

Content of	Group^1^ n = 10		SEM	*P*-value
	1	2		
Cholesterol	55.44	54.01	1.23	0.467
Collagen	28.95^A^	20.27^B^	1.37	<0.001

^1^ – 1 = feed based on soybean meal, 2 = feed based on faba bean (*Vicia faba* var. minor), n – number of samples taken to analysis.

A, B… – mean values marked in columns with different letters differ significantly between groups, *P*-value < 0.01.

## Discussion

Similar to other studies^2,21–23^ we found no significant effect of faba bean used as a substitute for soybean meal on the values of pH_24_ measured in breast and leg muscles. Conversely to our study, Laudadio *et al*.^21^ reported that the change of protein source in the diet influenced the colour of breast muscles. They found significantly lower values of lightness (L*) for breast muscles from chickens fed diet based on faba bean. In our study, the redness (a*) of breast muscles did not differ between the analysed feeding groups. As with the colour of leg muscles, Laudadio *et al*.^21^ reported differences in the values of redness (a*) and yellowness (b*), which were higher in chickens fed a diet with the inclusion of faba bean compared to chickens fed soybean meal. This was not confirmed in our study. Laudadio *et al*.^21^ suggested that higher values of yellowness (b*) in leg muscles could be attributed to different fatty acids composition and the S/P ratio in these muscles. A trend towards higher yellowness (b*) of leg muscles was also reported by Milczarek *et al*.^23^, who investigated the effect of various levels of low- and high-tannin faba bean on chicken meat. In our study we found significantly lower values of lightness (L*) for leg muscles from chickens fed a diet based on faba bean. This was not consistent with findings by Laudadio *et al*.^21^ and Milczarek *et al*.^23^. On the other hand, Dal Bosco *et al*.^22^ investigated the effect of faba bean (16% of ration), and reported, similar to our study, no significant influence of this feed component on the value of yellowness (b*) of leg muscles from chickens.

Consistently with our study, Milczarek *et al*.^23^ found no effect of faba bean (low- and high tannin, at various levels per feed ration) on the water holding capacity (WHC) of leg muscles. Laudadio *et al*.^21^ reported, unlike in our study, that the water holding capacity of breast and leg muscles differed depending on diet, and was higher in chickens fed faba bean. Nevertheless, different sources of protein in diets had no effect on drip loss from breast muscles, which was also confirmed in our study. Findings from studies investigating the effect of diets with faba bean on the chemical composition of muscles from broiler chickens are inconsistent. For example, Osek *et al*.^24^, Laudadio *et al*.^21^ and Milczarek *et al*.^23^ reported that the dietary inclusion of faba bean as a source of protein had no effect on the content of protein and fat in chicken breast and leg muscles. This was confirmed in our study. On the other hand, Meluzzi *et al*.^25^, Dal Bosco *et al*.^22^ and Osek *et al*.^24^ reported significantly lower content of fat in leg muscles from chickens fed balanced feed mixtures containing faba bean.

In our study breast muscles from broiler chickens fed a diet with the inclusion of faba bean contained more C16:0 fatty acid. Similar conclusions were reached by Laudadio *et al*.^21^, who found a higher content of palmitic acid and higher total content of saturated fatty acids (SFA) in breast muscles. They also reported higher total content of n-3 fatty acids in muscles from chickens fed faba bean compared to chickens fed soybean meal. This was associated with a significant increase in the content of n-3 fatty acids, including eicosapentaenoic acid (C20:5n-3), docosapentaenoic acid (C22:5n-3) and docosahexaenoic acid (C22:6n-3) in breast and leg muscles. The authors of the cited study suggested that the dietary inclusion of faba bean significantly reduced the content of monounsaturated fatty acids in breast and leg muscles. They also reported a reduced n-6 to n-3 ratio in breast and leg muscles from chickens fed faba bean. Our study did not confirm this, since the analysis of breast muscles revealed a significantly higher n-6 to n-3 ratio in chickens fed faba bean (12.43) compared to chickens fed soybean meal (8.74). The increase in this ratio could be attributed to significantly higher (P < 0.01) content of C22:4 n-6 fatty acid in chickens from group 2. In our study the dietary inclusion of faba bean as the source of protein had no effect on the values of AI and TI in breast muscles from broiler chickens. Similar conclusions were reached by Laudadio *et al*.^21^, but Milczarek *et al*.^23^ found lower values of AI and TI in chickens fed with different levels of high- and low- tannin varieties of faba bean. Ulbricht & Southgate^26^ suggested that lower values of AI and TI in muscles indicate a more beneficial effect of chicken meat on consumer health.

In our study the replacement of soybean meal with faba bean did not increase the content of cholesterol in chicken breast muscles, but was associated with reduced content of collagen in these muscles. Laudadio *et al*.^21^ also demonstrated that different sources of protein (soybean meal, faba bean) influenced the total content of collagen in breast and leg muscles. Conversely, our study revealed a higher total content of collagen in muscles from chickens fed faba bean compared to chickens fed SBM. Some differences between our study and cited authors could be caused by the other genotype of chickens that was used in experiments, or even various species (chickens, turkeys). The maintain conditions could be different, as well as the method of feed preparing, including cultivar of faba bean seeds.

The use of faba bean as a substitute for soybean meal had no significant effect on most physicochemical parameters and chemical composition (content of protein, fat and water) of breast and leg muscles from 6-week-old broiler chickens. Different sources of protein in feed mixtures for chickens had a slight effect on fatty acids composition and content of collagen in breast muscles. Nevertheless, dietary inclusion of faba bean did not increase the content of cholesterol, or values of AI and TI in breast muscles that would be important for consumer health. Overall, the use of faba bean as an alternative to soybean meal in the diet of broiler chickens had positive effects on meat quality. It is important, because it could be an alternative for small-scale farms, where feed for animals are from own crops.

## Methods

The slaughter of birds was carried out in accordance with the applicable rules on the handling of animals at the time of slaughter, including humane treatment. Also the methods used in the meat quality tests were carried out in accordance with the current and commonly used methodology described in the Material and methods section. Accordingly to the directive no. 2010/63/EU the approval of Ethics Committee was not required. The directive states that the requirements for the protection of animals used for experimental purposes. There it is described that these rules do not apply to agricultural activities and animal husbandry. The experiment was conducted in commercial conditions, so farmers were responsible for rearing. In addition, there is resolution 13/2016 of the National Ethics Committee for Animal Experiments of June 17, 2016 where: Collecting material from animals in breeding for genotyping and marking these animals are not procedures within the meaning of the Act on the protection of animals used for scientific or educational purposes and you do not need to obtain the consent of the Local Ethics Committee.

### Animals and diets

The study was conducted on 340 Ross broiler chickens divided into two subgroups, into 5 replications with 34 birds per each. Group 1 (control) received soybean meal as a source of protein, and group 2 (treatment) received faba bean. There were two feeding phases: days 1–14 and days 15–42 (Table 5). The content of concentrate in the diet during two feeding phases is presented in Table 5. Group 1 received concentrate with 78.89 to 76.37% content of soybean meal. Protein in the diet of group 2 was mainly sourced from faba bean, and its content was in the range of 51.22–52.13% (Table 6). Content of crude protein (CP) was at 170–230 g/kg dry matter feed and metabolic energy (ME) was at 12.60–13.45 MJ/kg of feed, according to recommendations by Smulikowska & Rutkowski^27^. Chickens were kept in pens on litter. Birds received feed and water ad libitum, and were reared for 6 weeks. The rearing period was conducted according to the commonly use recommendations of broiler chickens’ production technology.

**Table 5:** Proportion of concentrate and corn in feed mixture for chickens.

Days 1–14	CONCENTRATE	CORN
Control group (1)	45%	55%
Treatment group (2)	63%	37%
Days 15–42	CONCENTRATE	CORN
Control group (1)	43%	57%
Treatment group (2)	62%	38%

1 – feed based on soybean meal, 2 - feed based on faba bean (*Vicia faba* var. minor).

**Table 6:** Composition of starter and grower concentrates for chickens.

Components (%)^2^	Starter		Grower	
	group 1^1^	group 2^1^	group 1^1^	group 2^1^
Faba bean	—	51.22	—	52.13
Potato protein	—	9.05	—	8.23
Yeast	—	6.35	—	4.84
RSM	—	15.87	—	16.13
SBM	78.29	—	76.37	—
Soybean oil	11.11	10.63	13.72	12.58
Premix 1%	2.22	1.59	2.33	1.61
Monocalcium phosphate	4.18	2.33	4.05	2.23
Fodder chalk	0.91	0.84	0.60	0.55
Fodder salt	0.29	0.19	0.30	0.24
NaHCO3	1.00	0.76	1.05	0.71
L-lysine	0.89	0.41	0.72	0.26
DL-methionine	0.51	0.38	0.47	0.34
L-threonine	0.38	0.16	0.28	0.08
L-valine	0.22	0.14	0.12	0.03
L-tryptophan	—	0.06	—	0.05

Feed rations were done based on recommendations by Smulikowska and Rutkowski (2018).

Crude protein (CP): 170–230 g/kg DM (dry matter) of feed, ME (metabolic energy): 12.60–13.45 MJ/kg of feed.

^1^ – 1 - feed based on soybean meal, 2 - feed based on faba bean (*Vicia faba* var. minor).

^2^RSM – rapeseed meal, SBM – soybean meal.

### Slaughter and meat quality

After 6 weeks of rearing, 20 birds (10 from each group), of body weight close to the mean for the whole group, were slaughtered. The slaughter was carried out by cutting off the head (cutting the neck artery) and rapid bleeding, previously causing loss of bird awareness using an electric current method. Breast and leg muscles were dissected and analysed for quality traits. The pH value of breast muscles was measured 15 minutes post-mortem (pH_15_) and after 24 h cold storage at 2 °C (pH_24_). Both measurements were taken with a CX-701 pH-meter with a knife electrode (Elmetron, Poland). The colour of breast and leg muscles was analysed with a colorimeter (Konica Minolta, model CR400, Japan), calibrated using the white calibration plate no. 21033065 and the D65Y86.1X0.3188y0.3362 scale. The colour was graded according to the CIE system for L* (lightness), a* (redness), and b* (yellowness)^28^. Drip loss from breast muscles was measured. Breast muscles were weighed post-mortem (M1) and after 24-hour cold storage at 2 °C (M2). Analysis was performed using a method by Honikel^29^. The water holding capacity of breast and leg muscles was analysed using a modified method by Grau & Hamm^30^. Pooled samples of disintegrated muscles (about 0.300 g) were wrapped in Whatman grade 1 filter paper and kept under 2 kg pressure for 5 minutes. The water holding capacity of meat was calculated based on the difference in weight before and after the test. Pooled samples of disintegrated breast and leg muscles (90 g) from each group were analysed for the content of protein, fat and water according to the PN-A-82109: 2010^31^ standard with FoodScan apparatus (FOSS), using Near InfraRed Transmission (NIT) spectrometry calibrated for an artificial neural network (ANN). Breast muscles were also analysed for fatty acids composition, and content of cholesterol and collagen.

### Fatty acid composition

Lipid extraction from breast muscle was performed^32^. Fatty acids (FA) were quantified as methyl esters (FAME) using a gas chromatograph GC Trace 2000 (ThermoQuest EC Instruments) with a flame ionization detector (260 °C) and a fused-silica capillary column (Zebron ZB-88, Phenomenex, Torrance, CA, USA) 100 m × 0.25 mm ×0.20 μm foil thickness. Helium as carrier gas was used. The temperature in owen was set at 100 °C for 5 min, then increased at 4 °C/min up to 240 °C and maintained for 30 min at 240 °C. The peaks for each individual fatty acid were identified by comparison of retention times with those of FAME authentic standards run under the same operating conditions. Results were expressed as the percentage of the total FA identified. The ratio of n-6 to n-3 FA (n-6/n-3) and the ratio of PUFA to SFA (P/S) were calculated. Moreover, the atherogenic index (AI) and the thrombogenic index (TI) were calculated^26^. Method used for fatty acid composition were done in the same way as it was described by Tavaniello *et al*.^33^.

### Cholesterol content

Cholesterol was extracted^34^ and then quantified by HPLC. A Kontron HPLC (Kontron Instruments, Milan, Italy) model 535, with a Kinetex C18 reversed-phase column (150 ×4.6 mm ×5 µm; Phenomenex, Torrance, CA) was used. The HPLC mobile phase consisted of acetonitrile: 2-propanol (55:45, vol/vol) at a flow rate of 1.0 mL/min. The detection wavelength was 210 nm. The quantitation of cholesterol content in muscles was based on the external standard method using a pure cholesterol standard (Sigma, St. Louis, MO). This method was also done according to the method of Tavaniello *et al*.^33^.

### Collagen content

The description of collagen content in breast muscle was done according to Maiorano *et al*.^35^. Muscle samples were thawed at room temperature, trimmed of fat and epimysium, lyophilized for 48 h, and hydrolyzed in Duran glass tubes (Schott AG, Mainz, Germany) in 5 ml of 6N HCl at 110 °C for 18 to 20 h^36^ for the determination of hydroxyproline^37^. The analyses were carried out in duplicate. Intramuscular collagen concentration was calculated assuming that collagen weighed 7.25 times the measured hydroxyproline weight^38^ and expressed in micrograms of hydroxyproline per milligram of lyophilized tissue.

### Statistical evaluation

Numerical data were processed with STATISTICA 10.0 PL software^39^. Means (x) and standard error of measurement (SEM) were calculated for each parameter using one-way analysis of variance (ANOVA). Means were done from each replications for both groups. The significance of differences was verified with the post-hoc Scheffe test, at significance level p-value < 0.05. Differences between groups were statistically significant when p-value was less than 0.05. Each chosen bird was taken as an fundamental unit to calculate mean values from each groups.

#### Ethics

The research were done with recommendations of directive no. 2010/63/EU. The approval of Ethic Committee was not required. The slaughter of birds was carried out in accordance with the applicable rules on the handling of animals at the time of slaughter, including humane treatment. Also the methods used in the meat quality tests were carried out in accordance with the current and commonly used methodology described in the Material and methods section.
